# Accurate and exact CNV identification from targeted high-throughput sequence data

**DOI:** 10.1186/1471-2164-12-184

**Published:** 2011-04-12

**Authors:** Alex S Nord, Ming Lee, Mary-Claire King, Tom Walsh

**Affiliations:** 1 Department of Genome Sciences, University of Washington, 1959 NE Pacific Street, Seattle, WA, 98195-7720, USA; 2 Department of Medicine, University of Washington, 1959 NE Pacific Street, Seattle, WA, 98195-7720, USA

## Abstract

**Background:**

Massively parallel sequencing of barcoded DNA samples significantly increases screening efficiency for clinically important genes. Short read aligners are well suited to single nucleotide and indel detection. However, methods for CNV detection from targeted enrichment are lacking. We present a method combining coverage with map information for the identification of deletions and duplications in targeted sequence data.

**Results:**

Sequencing data is first scanned for gains and losses using a comparison of normalized coverage data between samples. CNV calls are confirmed by testing for a signature of sequences that span the CNV breakpoint. With our method, CNVs can be identified regardless of whether breakpoints are within regions targeted for sequencing. For CNVs where at least one breakpoint is within targeted sequence, exact CNV breakpoints can be identified. In a test data set of 96 subjects sequenced across ~1 Mb genomic sequence using multiplexing technology, our method detected mutations as small as 31 bp, predicted quantitative copy count, and had a low false-positive rate.

**Conclusions:**

Application of this method allows for identification of gains and losses in targeted sequence data, providing comprehensive mutation screening when combined with a short read aligner.

## Background

Massively parallel sequencing technology can be used to efficiently interrogate multiple targeted genomic regions for clinically relevant mutations [1]. The use of short unique sequence indexes, or barcodes, increases sequencing throughput and allows analysis of multiple samples in a single sequencing run. Application of this technology will allow cost-efficient screening of genes known or suspected to harbor clinically relevant pathogenic mutations. While small mutations are readily detectable with current analysis platforms [2,3], methods for the identification of copy-number variants (CNVs) are not well established for targeted data. The ability to detect the full spectrum of mutations is critical to the success of targeted sequencing projects.

Current methods for detecting structural variation from massively parallel data use either paired-end mapping or depth of coverage methods (see [4] for a recent review). Paired-end mapping methods perform well for mate-pair sequence data and have the advantage of identifying both balanced (e.g. translocations or inversions) and unbalanced (deletions and duplications) structural variation. Comprehensive CNV scanning using paired-end mapping requires mulitple insert sizes and the presence of paired tags that bridge the breakpoint of the event. This approach is widely used in the assembly of whole genomes but cannot be applied to targeted enrichment protocols, as hybridization-based capture methods for massively parallel sequencing typically require small insert fragments (200 bp)[5] and breakpoints of structural variants may lie outside of targeted regions.

Depth of coverage methods do not require breakpoint capture or have specific size restrictions, yet such methods are hampered by low signal to noise ratio due to sparse sampling (a product of low overall coverage) and sampling bias based on sequence GC-content, bait coverage, and other factors affecting capture efficiency. Further, depth of coverage methods require cross-sample normalization and comparison in order to deal with variation in coverage across genomic regions and samples. We previously showed that a basic depth of coverage approach performs well to identify CNVs from high-coverage (~1000×) targeted sequence data[1]. Depth of coverage methods have achieved higher signal to noise by averaging data across a genomic region[6-8], however, doing so results in a loss of the ability to detect smaller mutations. In addition, depth of coverage methods are poor at localizing breakpoints [4].

Here we describe a method that combines CNV detection through high-resolution depth of coverage analysis with call confirmation through partially-mapped reads. This allows for the identification of DNA gain or loss even where breakpoints are outside the targeted regions, and exact breakpoint characterization where breakpoints are within the targeted regions. By combining these orthologous approaches, CNVs of any size can be identified from targeted high-throughput sequence data with a low false-positive rate.

## Results

Our method uses two independent approaches, depth of coverage and a scan for partially-mapped reads at CNV edges. We implemented a straightforward depth of coverage algorithm and internally validated CNV calls by looking for a signature of partially-mapped reads that confirm the CNV breakpoints where possible. Partially-mapped reads are defined as high-quality full length (76 bp) reads where the best alignment to targeted regions is a perfect match less than 76 bases in length. A minimum match length of 30 bp was used for this analysis. Where partially-mapped reads match to a CNV edge, it is assumed that the unaligned portion of the read maps to sequence flanking the other breakpoint and the partially-mapping reads can be aligned to each other to form a consensus sequence that flanks the breakpoint. For example, in the case of a deletion, the partially mapped reads will correspond to sequence flanking the deleted region, giving the exact breakpoints of the CNV. Partially-mapped reads map to the reference sequence differently for gains and losses, as described for paired-end mapping methods of CNV detection [4]. Where CNV breakpoints lie within non-targeted sequence, partially-mapped reads are unavailable and depth of coverage is the only evidence used for CNV calling.

**Figure 1:**
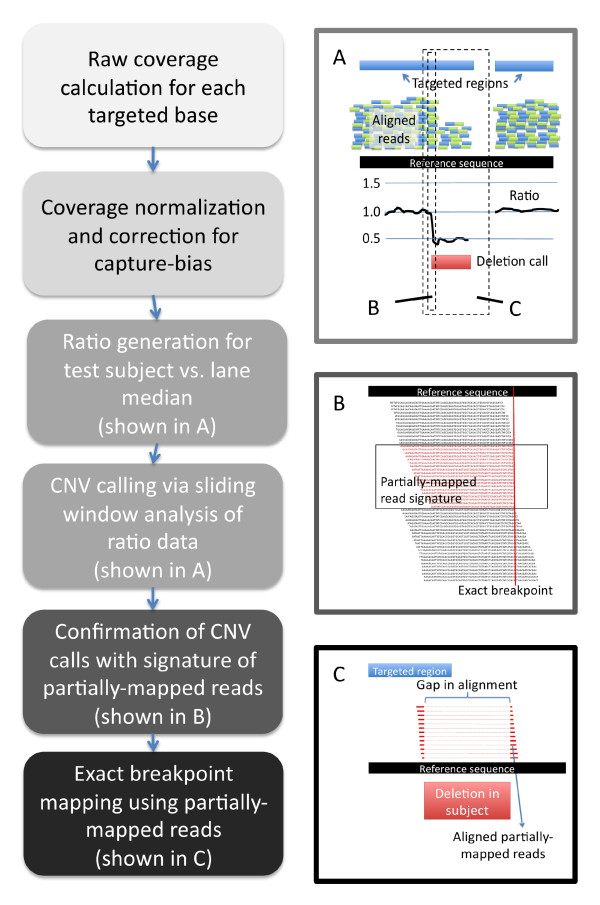
**Analysis Schema**. The left side of the figure shows a flow chart summarizing our methods. The right panels illustrate specific processes in the identification of a hemizygous deletion where one edge of the CNV is within targeted sequence. **a) **Targeted region with short reads aligned to the reference sequence, ratio of normalized coverage for sample versus lane median, and CNV call based on depth of coverage analysis. **b) **Sequence reads that align to the CNV edge shown in a). Black reads align across the complete 76 bp. Red reads are shorter segments of 76 bp reads that align perfectly to the region and indicate presence of mutation. **c) **Reads that partially-map to the CNV edge will also align to sequence flanking the other edge and can be used for exact breakpoint characterization, despite the one edge being in non-targeted sequence. The gap in the alignments represents the deleted sequence.

Figure 1 outlines the method we describe here for calling CNVs and identifying exact breakpoints using sequence map data. The analysis schema is shown on the left side of the figure. The right side depicts the processes using a hemizygous deletion with one breakpoint in targeted sequence and the other in flanking non-targeted sequence as an example. For each sample, raw coverage counts are generated and then normalized to the median of the other samples in the lane using invariant set methods^5^. Normalized coverage is further corrected for sample-specific capture bias due to GC-content and bait probe hybridization likelihood. Sample versus median ratio is then derived, and CNVs are called using a sliding window method to identify regions with ratio values below 0.6 or above 1.4 (Figure 1, Panel A). Depth of coverage CNV calls are confirmed where possible by examining CNV edges for a signature of partially-mapped reads (Figure 1, Panel B). Partially-mapped reads that capture a breakpoint in unique targeted sequence are aligned to form a consensus sequence which is then mapped back to the genome to identify exact breakpoints (Figure 1, Panel C).

**Figure 2:**
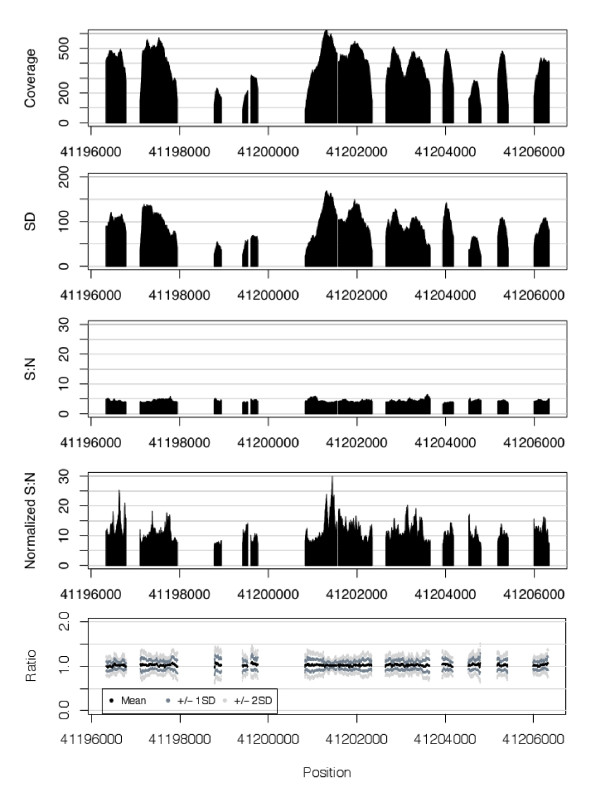
**Raw and normalized coverage data**. Data for region of *BRCA1 *on chr17 where all samples represented are diploid. Mean and standard deviation (SD) for raw coverage across one lane (12 subjects) shown in top two panels. The third and fourth panels show signal-to-noise ratio for the raw and normalized data. Signal-to-noise was calculated as mean/SD for each base. The final panel shows mean and standard deviation for the ratio data from the 12 individuals across the region.

Our data were massively parallel sequence reads from a capture design that targets genomic sequence across 21 genes which are known to predispose to high risks of breast and ovarian cancer[1]. A total of 909 kbp sequence was targeted in 96 samples concurrently, running 12 samples per lane on an Illumina GAIIx instrument. Sequencing was performed with a standard multiplex 2 × 76 paired-end reads using TruSeq SBS chemistry and analyzed with Illumina SCS2.8/RTA 1.8 and the demultiplexing script within CASAVA http://www.illumina.com. Single base coverage was calculated based on the number of tags for each sample overlapping each base across targeted regions. Figure 2 illustrates the regional variation in raw coverage and the increase in signal-to-noise ratio after cross-sample normalization for a representative diploid region of *BRCA1*, resulting in ratio values that closely cluster around one in a region with no CNVs in the samples analyzed.

We obtained median raw coverage of 350× across the 909 kbp of targeted sequence (Additional file 1, Figure S1). Low coverage regions were primarily GC-rich, consistent with other targeted sequence data[9], and account for the majority of bases excluded from screening for structural variation. 99.8% of targeted bases had median coverage of at least 10×, permitting high-confidence detection of single base and short indels for nearly all sequence assayed. In order to estimate the effect of random noise on the false-negative rate, we simulated data where one CNV of 50 bp, 100 bp, 200 bp, 500 bp, or 1000 bp was present within 1 mb of sequence and tested for detection of the CNV across varying signal-to-noise ratios (Additional file 2, Figure S2). This analysis indicated that our methods can detect a 200 bp CNV with 87% sensitivity at a signal-to-noise ratio of 6, a criteria met by >92.3% of the bases targeted in this experiment, and a 100 bp CNV with 80% sensitivity at signal-to-noise ratio of 8, a criteria met by 75.8% of the bases.

**Figure 3:**
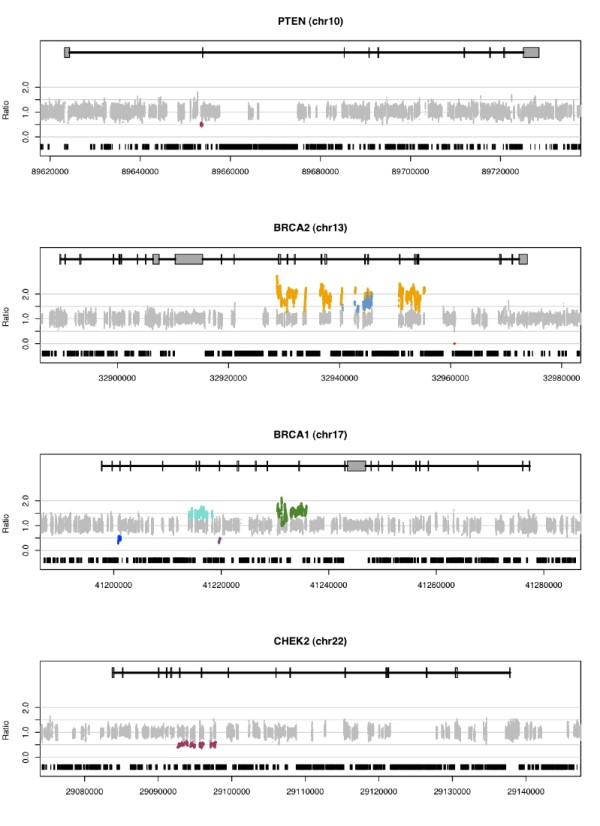
**Ratio of sample to median corrected depth of coverage indicates variant regions**. Each subplot shows ratio across one targeted region (*PTEN, BRCA2, BRCA1*, and *CHEK2*), with CNVs shown as colored datapoints. Using depth of coverage with map confirmation, we identified 10 CNVs (5 deletions (one homozygous), 4 duplications, and 1 triplication) across 21 targeted regions (909 kbp) for 96 barcoded samples. CNV size ranged from 31 bp to 26560 bp. Ratio calculated by comparing corrected normalized sample coverage to median coverage within one flow cell lane. Diploid bases are plotted in grey, while colored datapoints indicate copy-number variant bases for one sample. Non-targeted repeat sequence is shown in black at bottom of each plot.

In the samples that passed quality control (94/96), we identified a total of 10 CNVs (5 gains and 5 losses) in four genes through depth of coverage analysis that passed minimum coverage and z-score requirements and were confirmed by Sanger sequencing or MLPA (Figure 3 and Table 1). For losses, one homozygous deletion and four hemizygous deletions were detected, with no reads from the homozygous deletion and median ratio across hemizygous deletion regions between 0.49 and 0.51. For single copy gains, median duplication ratios were between 1.44 and 1.62. A triplication was also observed with a ratio of 1.90, demonstrating that ratio accurately reflects copy number. All samples were previously tested for CNVs in *BRCA1 *and *BRCA2*, and we correctly identified all known mutations and generated no false positive calls in these genes.

**Table 1:** CNVs identified in 94 subjects across ~1 Mb targeted sequence

Sample_ID	Region	Chr	Start	End	Class	Size	Call	Targeted bases	Median Ratio	Median Z-score	Median S:N	Median Sample Coverage	Median Lane Coverage
CF175_01	PTEN	chr10	89652825	89653723	Deletion	899	DoC + Seq	281	0.45	-8.58	15.58	196	519
CF682_01	PTEN	chr10	89652825	89653723	Deletion	899	DoC + Seq	281	0.51	-8.63	17.04	148	522
CF1815_01	BRCA2	chr13	32928730	32955289	Triplication	26560^a^	DoC	10560	1.90	11.16	12.11	649	359
CF804_17	BRCA2	chr13	32940522	32945860	Duplication	5339^a^	DoC	1702	1.63	6.13	10.15	318.5	272
CF815_02	BRCA2	chr13	32960705	32960735	Homozygous Deletion	31	DoC + Seq	31	0^b^	na	11.94	0	249
CF605_01	BRCA1	chr17	41200740	41201249	Deletion	510	DoC + Seq	419	0.49	-7.79	14.98	119	312
CF456_01	BRCA1	chr17	41214000	41218359	Duplication	4360^a^	DoC	2332	1.47	7.06	15.45	277	368
CF163_02	BRCA1	chr17	41219555	41219794	Duplication	240^a^	DoC	240	0.43	-5.26	9.11	77	264
CF499_01	BRCA1	chr17	41230471	41235875	Duplication	5405^a^	DoC	4000	1.55	8.35	15.93	391	429
CF682_01	CHEK2	chr22	29092633	29099332	Deletion	6700^a^	DoC	2570	0.50	-8.16	16.21	108	388

^a ^Size reflects minimum size, as breakpoints are located within flanking repetetive sequence

^b ^For homozygous deletion, no reads are present within region

CNVs identified within high-risk breast and ovarian cancer genes targeted by this experiment. Calls were made via depth of coverage (DoC) and confirmed with partially-mapped read analysis (Seq) where possible.

**Figure 4:**
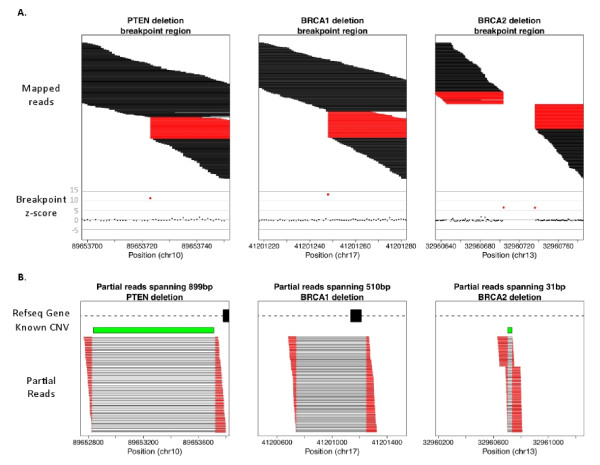
**Use of mapped partial reads to confirm calls and identify exact breakpoints**. We tested for over-representation of tag start or end across predicted CNV breakpoint, and then mapped partial reads to identify exact breakpoints. We confirmed an 899 bp *PTEN *deletion present in two samples, a 510 bp deletion in *BRCA1*, and a 31 bp homozygous deletion in *BRCA2* using this method. **a) **Unique breakpoint region for each CNV with sequence tags plotted by start and end position. Tags where all 76 bases align are shown in black, and tags where less than 76 bases align are shown in red. Z-scores generated based on the number of reads that start or end at each base are shown below the mapped reads with red indicating breakpoint(s). **b) **Each read that partially maps to the breakpoint aligns to sequence flanking the other side of the CNV, allowing exact breakpoint identification. Partial reads are shown in red, with a line connecting the two segments of each read. Length for all reads shown is 76 bp.

After applying depth of coverage methods to detect CNV regions, we mapped sequence reads at the edges of the events to confirm the calls and identify exact breakpoints. This process requires that at least one breakpoint be within unique targeted sequence, as targeted capture generates sequences only over targeted regions and non-unique short sequence tags are difficult or impossible to map accurately. Of the 10 CNVS, four deletions were predicted to have at least one breakpoint in unique sequence. Using a signature of partially-mapped reads, we confirmed these calls and identified exact breakpoints for all four events (Figure 4). While we identified no duplications with edges in unique sequence, duplications will produce a similar signature to deletions and can be confirmed using the same process.

The first deletion, of 510 bp, removes an exon of *BRCA1 *(chr17:41,200,740-41,201,249). We confirmed a 31 bp homozygous deletion in *BRCA2 *(chr13:32,960,705-32,960,735) that matches a known indel (rs56213495), indicating that our method is sensitive to small CNVs. The final two deletions mapped to the same *PTEN *coordinates and overlap with a known CNV previously identified by array comparative genomic hybridization (aCGH) (DGV ID: 657536[10]). In comparison to aCGH methods, we were able to resolve exact breakpoints for this 899 bp polymorphic deletion (chr10:89,652,824-89,653,723). Importantly, mapping sequence tags to the breakpoints allowed exclusion of a large number of false positive calls. All seven CNVs with breakpoints predicted to be within repetitive non-targeted sequence were validated by Sanger sequencing or MLPA. In the 909 kbp in 94 samples there was one CNV called that could not be confirmed. This 109 bp duplication could not be verified through sequence mapping or Sanger sequencing and is a likely false positive. Breakpoint resolution via partial read mapping reduced the number of false positives to a single duplication across 94 samples and 909 kbp.

## Discussion

Targeted high-thoughput sequencing data is used to produce high-coverage (often >50×) sequence data on specific regions of the genome. Targeted regions are often non-contiguous, as in the case of coding sequence capture or interruptions due to repeat sequences. We created a method that is specific to high-coverage, non-contiguous sequence data for robust CNV detection from targeted sequence data. Our method does not require sequencing across CNV breakpoints (as paired-end methods do), but leverages sequence level data for exact CNV characterization where breakpoints are within targeted regions. Using a test dataset of regions that contribute to inherited breast and ovarian cancer susceptibility, we identified 10 mutations (7 known to be pathogenic and 3 benign), localizing 4 mutations to exact genomic breakpoints. The minimum size for a CNV that we detected was 31 bp, and there was only one suspected false positive among all samples that passed quality control.

Our method has several advantages over previous methods used to detect CNVs from high-throughput sequencing. While sequence-based CNV signal (e.g. partially-mapped reads or split paired-end reads) is preferable over relative coverage, sequence data is unavailable in targeted data whenever the CNV breakpoints are not in the targeted region. This introduces a major issue for methods that rely on sequence-based data for CNV detection (e.g. [11,12]). Additionally, as described earlier, paired-end mapping strategies are not currently applicable when targeted enrichment is used. In comparison, relative depth of coverage is problematic due to local variation in coverage within samples and variation in coverage across samples. This leads to problems with false-positive signal when scanning for regions that depart from expected diploid coverage. This issue can be partially addressed by averaging read depth across a genomic region (e.g. [6-8]), however, doing so reduces the ability to detect small mutations. By combining ortholgous algorithms, we eliminated from consideration a large number of false-positive CNVs called by depth of coverage methods where no signal of partially-mapped reads was present. The ability to computationally resolve the majority of false-positive calls permitted us to use high-resolution analysis even where signal-to-noise ratio was intermediate. In addition, depth of coverage analysis has the benefit of producing a quantitative estimate of copy-count, as demonstrated by the clear distinction between duplication and triplication ratios.

The major limitation of depth of coverage methods arises in duplicated genomic regions, such as segmental duplications or smaller regions of homology between genomic regions. Within such regions, the expected ratio for deletions and duplications is relative to the copy count in the genome. For depth of coverage, a copy number change from two to one (hemizygous deletion) or two to three (duplication) produces a strong signal, however, copy number changes in duplicated regions of the genome produce weaker signal that can be easily overwhelmed by noise. For example, variation between samples will overwhelm the signal of a change from six copies to five, where the expected ratio would be 0.83. As such, we limit analysis to unique regions of the genome. In addition, depth of coverage methods do not detect balanced structural variation.

The methods we describe here were optimized for multiplexed high-coverage data, but we expect the fundamental approach will be applicable to experiments with a larger volume of targeted sequence, such as exome capture. While we used multiplexed data from a relatively small number of targeted bases, the method we describe is scalable and could be used on any dataset where signal-to-noise ratio allows reliable depth of coverage CNV calling. Where local variation is too high or coverage too low, average coverage across a genomic window can be used instead of single base coverage, although this lowers resolution to the size of the window used. However, scanning across the window for partially-mapped reads as we do here would still permit exact CNV breakpoint characterization when breakpoints are in targeted sequence.

## Conclusions

Targeted massively parallel sequencing permits screening of genomic regions for multiple samples simultaneously, and thus is a powerful and cost-effective tool for characterizing mutations in contexts where multiple genes or pathways are involved[1,13-15]. We demonstrate that a combination of depth of coverage and analysis of partially-mapped reads allows detection with high-confidence of CNVs within unique sequence, to a resolution of 31 bp. Furthermore, we demonstrate that ratio can be used to estimate absolute copy number and that depth of coverage alone is sufficient for CNVs where breakpoints are not represented within targeted regions. The method we describe here is robust, yet simple to implement; while we employ specific mapping and segmentation algorithms, the overall framework is amenable to improvement and variation in the algorithms as massively parallel sequencing technology progresses.

## Methods

Sample selection and DNA extraction were as described previously[1]. Library preparation, sequencing, and mapping of reads to the genome performed as described per Illumina and Agilent protocols http://www.illumina.com and http://www.agilent.com. Three micrograms of high quality genomic DNA was sonicated on a Covaris S2 to a peak size of 150-200 bp. DNA was end-repaired, A-tailed, ligated to standard Illumina adapters and amplified with flanking primers. 500 ng of paired-end library was hybridized individually to a custom design of cRNA oligonucleotides ([9]Agilent, ELID:0279281, BED file available upon request) and amplified with index specific primers from the Illumina Mutiplex Oligonucleotide kit. Equal concentrations (1.3 pM) of 12 paired end indexed libraries were pooled per lane on a v4 flow cell and subjected to cluster amplification on a cBot instrument. Raw sequence coverage was determined by tabulating the number of tags that overlapped each targeted base as mapped by the standard Illumina GAIIx bioinformatics pipeline with the demultiplexing script http://www.illumina.com. Coverage data for the targeted regions is shown in Additional file 3, Table S1.

The computational methods described here can be separated into four steps: raw coverage normalization, correction for sample-specific coverage biases, CNV calling, and partially-mapped read analysis. All analysis was done using the R platform http://www.r-project.org with custom scripts, which are available at request. We emphasize that the methods described below are one specific implementation within a general framework. The approach we describe is amenable to alternative normalization methods, application of any segmentation algorithm that can employ ratio data, and use of other sequence mapping algorithms suitable for short read data.

### Coverage normalization

Single sample coverage was normalized to the other eleven samples run in the same lane. Normalization was performed using invariant set methods[16]. Briefly, bases that had a similar coverage rank in the sample and median data were selected and a line was fit using a smooth spline function across the range of coverage counts. Normalization reduced the median standard deviation from 85.7 for raw coverage to 27.1 after normalization (Additional file 1, Figure S1).

### Correction for GC-content and bait capture bias

Normalized coverage was further corrected for capture bias associated with GC-content and bait probe capture likelihood. GC-based effects on capture efficiency may differ across samples, as has been observed for array based hybridization methods[17]. After testing multiple window sizes (25 bp, 50 bp, 100 bp, 200 bp, and 500 bp), we found the strongest association between the range of coverage variation and GC-content in the 100 bp surrounding each targeted base. In preliminary data, we observed a parabolic relationship between GC-content and coverage with an apex around .4. This analysis applies to the specific sequencing platforms used for this experiment and GC-based parameters and strength of effect are likely platform dependent. In order to address GC-based bias in our data, we transformed GC-content using the equation:

where gc is the proportion of G/C bases within the 100 bp window surrounding a given base. Bait capture likelihood is associated with the chance of stable hybridization of the library insert (150-200 bp for our experiment) to bait probes targeting the insert. Our capture design used 3× coverage tiled every 40 bp with 120 bp bait probes. Use of a 40 bp tiling step resulted in true 3× coverage from base 80 to n-80, where n is the length of each contiguous targeted region. Bases 1 to 40 and n-40 to n had 1× tiling and 40-80 and n-80 to n-40 had 2× coverage. Coverage increases logarithmically from the first captured base to the middle of the contiguous targeted region. The first captured base can be outside the region of bait-probe hybridization. Based on preliminary data, we used a simple logarithmic transformation to account for this:

where d is the distance from the first targeted base and the constant 10 is used to account for off-target capture where the first captured base is outside of the region of hybridization.

Normalized coverage was corrected using simple linear regression with the transformed GC-content and bait capture likelihood terms as the independent variables and log(normalized coverage) as the dependent variable. Bases with coverage < 50 were excluded from contributing during curve fitting, as these bases show high levels of variation due to sparse sampling effects. Signal to noise ratio (mean/standard deviation) went from 12.3 for uncorrected normalized coverage, to 13.2 after correction (Additional file 1, Figure S1). 99% of bases had corrected coverage signal to noise ratio > 5 and 83% had signal to noise ratio > 10. Bases with signal to noise < 2 were excluded from CNV analysis.

### Depth of coverage CNV calling

Ratio data was calculated by comparing sample corrected coverage to the median corrected coverage of the other 11 samples in the lane. We used a sliding window to identify regions where the majority of bases had a ratio < = 0.6, indicating copy number loss, or > = 1.4, indicating copy number gain. Window size was 20 bp, with a minimum of 18 bp required to meet the criteria for either gain or loss. Copy-number variant regions were generated by extending variant windows and merging neighboring variant regions. The median z-score across the CNV region was calculated by comparing sample ratio to mean and standard deviation of the remaining 11 samples in the lane. Z-scores closer to zero could reflect either diploid regions with high variation in coverage across samples or regions with an underlying common copy number polymorphism. As we were interested in rare mutations, CNV regions were required to meet the criteria of median absolute z-score > = 5 and signal to noise ratio > = 9 for acceptance. These values were set based on the minimum z-score and signal to noise ratio for CNVs that could be confirmed with Sanger sequencing.

Across diploid regions (where no CNV was both called and confirmed), 98.9% of the ratio values were between .6 and 1.4 (Additional file 1, Figure S1), compared to only 9.3% for confirmed CNV regions. The majority of bases that had ratio values < = 0.6 or > = 1.4 represented false positive signal, as no CNV could be confirmed. However, these false positive bases showed low signal to noise and z-scores were closer to zero versus true positive bases (Additional file 4, Figure S3). For ratio values for regions where a deletion was present and ratio values were < 0.6, 89.2% had signal to noise values > 10 and 91.1% had z-score values < -5. For regions with a confirmed duplication and ratio values > = 1.4, 78.6% had signal to noise values > 10 and 91% had z-scores > 5. The differences in signal to noise and z-score between confirmed CNV bases and false positive CNV bases indicates that ratio is a stronger indicator of true copy number when signal variation is taken into account.

### Partial read mapping

CNVs identified using depth of coverage methods were then examined for the presence of partially-mapped reads within the predicted breakpoint region. This is only possible for CNVs where at least one breakpoint is within targeted sequence. CNVs where both breakpoints appear to be in non-targeted regions (e.g. repeat sequence or sequence outside the targeted region boundaries) were excluded from this step. Repeat regions often mediate CNV formation[18-20] and minimum contiguous targeted region size was limited by bait probe size to 80 bp in this experiment, the majority of calls where both breakpoints are in repeats were confirmed by Sanger sequencing (7/8) and all calls were >100 bp in length, indicating that such calls are likely to be true positives. For calls where at least one breakpoint is unique we used BLAST[21] to test for partial reads that aligned to the specific breakpoint region. Partial reads were 76 bp in length and mapped at least 30 bp to the region. These parameters were selected as 76 bp is a full length read, and 30 bp was set as minimum cut-off to avoid mapping repeat sequence fragments. These lengths are dependent on platform read length and should change accordingly. Mean and standard deviation were calculated for the number of partial reads across the targeted breakpoint region, and z-score for each base was calculated and used to detect breakpoint based on over-representation of tags with the same start/stop position. Where no over-representation of start/end sites for partial reads was present, CNV calls were considered to be false positives. If a signature of partial reads was present, we mapped the consensus sequence of aligned partial reads to the genome to identify the exact CNV breakpoints.

## Competing interests

The authors declare that they have no competing interests.

## Authors' contributions

ASN, ML, TW, and M-CK designed the experiment. ASN designed the computational methods with assistance from ML. ASN, TW, and M-CK. drafted the manuscript. All authors read and approved the manuscript.

## Supplementary Material

Additional file 1 **Figure S1. Histograms showing distributions for count and variation of coverage and ratio data**. **A) **Median raw coverage: median coverage across samples for each base. **B) **SD raw coverage: standard deviation for raw coverage generated for each lane (8 samples), median SD across 8 lanes plotted. **C) **SD normalized coverage: standard deviation for normalized coverage generated for each lane (12 samples), median value across 8 lanes plotted. **D) **S:N normalized coverage: signal to noise ratio (mean/SD) for normalized coverage for each lane, median value across 8 lanes plotted. **E) **S:N corrected coverage: signal to noise ratio (mean/SD) for normalized coverage corrected for GC-content and bait capture bias for each lane, median value across 8 lanes plotted. **F) **Ratio: sample compared to lane median corrected normalized coverage for all bases, data from 10 representative samples plotted. Click here for file

Additional file 2 **Figure S2. Simulated sensitivity estimate based on CNV size and signal-to-noise ratio of data**. Data simulated for 1 mb of sequence with one true CNV of length 50 bp, 100 bp, 200 bp, 500 bp, or 100 bp. Random noise introduced in sample coverage data at a level corresponding with given signal-to-noise ratio. 100 replications run at signal-to-noise ratios of one to ten for each CNV size. Sensitivity is the proportion of runs in which the CNV was correctly identified. No false positives were identified when signal-to-noise ratio was greater than two (data not plotted). Click here for file

Additional file 3 **Supplemental table S1**. Click here for file

Additional file 4 **Figure S3. Comparison of true positive signal and false positive signal**. True positive refers to bases within confirmed CNVs, whereas false positive refers to bases with ratio values < 0.6 or >1.4, but where no CNV could be confirmed. Histograms show distribution of: **A) **S:N (signal to noise: mean/SD), and **B) **z-score ((value-mean)/SD) for true positive versus false positive bases. Click here for file
