# Supplementary material for: Accurate and exact CNV identification from targeted high-throughput sequence data
Source: BMC Genomics. 2011 Apr 12;12:184. doi: 10.1186/1471-2164-12-184 (PMC3088570; doi:10.1186/1471-2164-12-184)
Supplement: Additional file 1 — Figure S1. Histograms showing distributions for count and variation of coverage and ratio data. A) Median raw coverage: median coverage across samples for each base. B) SD raw coverage: standard deviation for raw coverage generated for each lane (8 samples), median SD across 8 lanes plotted. C) SD normalized coverage: standard deviation for normalized coverage generated for each lane (12 samples), median value across 8 lanes plotted. D) S:N normalized coverage: signal to noise ratio (mean/SD) for normalized coverage for each lane, median value across 8 lanes plotted. E) S:N corrected coverage: signal to noise ratio (mean/SD) for normalized coverage corrected for GC-content and bait capture bias for each lane, median value across 8 lanes plotted. F) Ratio: sample compared to lane median corrected normalized coverage for all bases, data from 10 representative samples plotted. [file 1471-2164-12-184-S1.PDF]

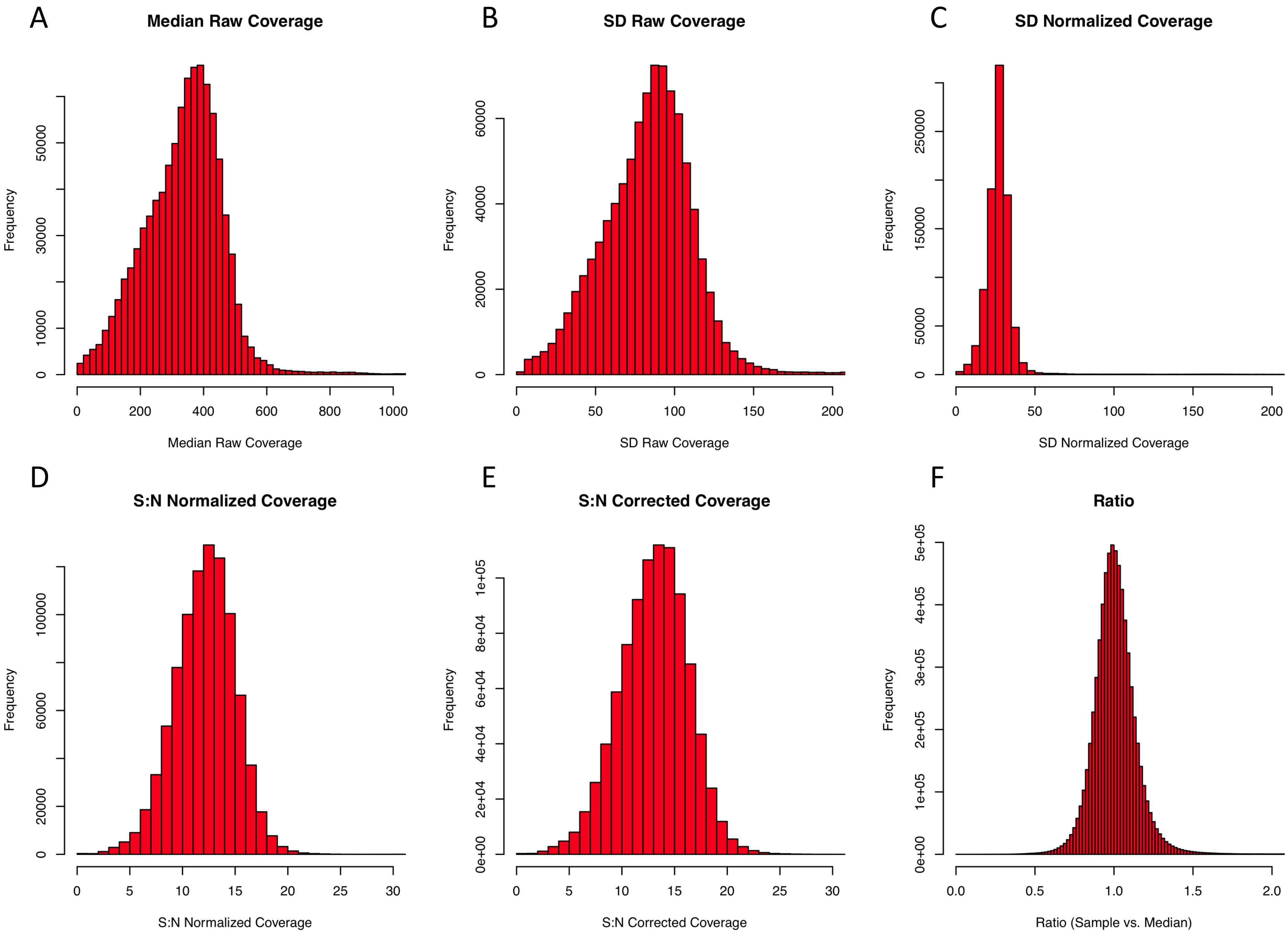

A
Median Raw Coverage
Frequency
0
10000
30000
50000
0
200
400
600
800
1000
Median Raw Coverage
B
SD Raw Coverage
Frequency
0
20000
40000
60000
0
50
100
150
200
SD Raw Coverage
C
SD Normalized Coverage
Frequency
0
50000
150000
250000
0
50
100
150
200
SD Normalized Coverage
D
S:N Normalized Coverage
Frequency
0
20000
60000
100000
0
5
10
15
20
25
30
S:N Normalized Coverage
E
S:N Corrected Coverage
Frequency
0e+00
2e+04
4e+04
6e+04
8e+04
1e+05
0
5
10
15
20
25
30
S:N Corrected Coverage
F
Ratio
Frequency
0e+00
1e+05
2e+05
3e+05
4e+05
5e+05
0.0
0.5
1.0
1.5
2.0
Ratio (Sample vs. Median)
