# Supplementary figures and images for: Accurate and exact CNV identification from targeted high-throughput sequence data
Source: BMC Genomics. 2011 Apr 12;12:184. doi: 10.1186/1471-2164-12-184 (PMC3088570; doi:10.1186/1471-2164-12-184)

# Effect of coverage signal to noise ratio (S:N) on sensitivity

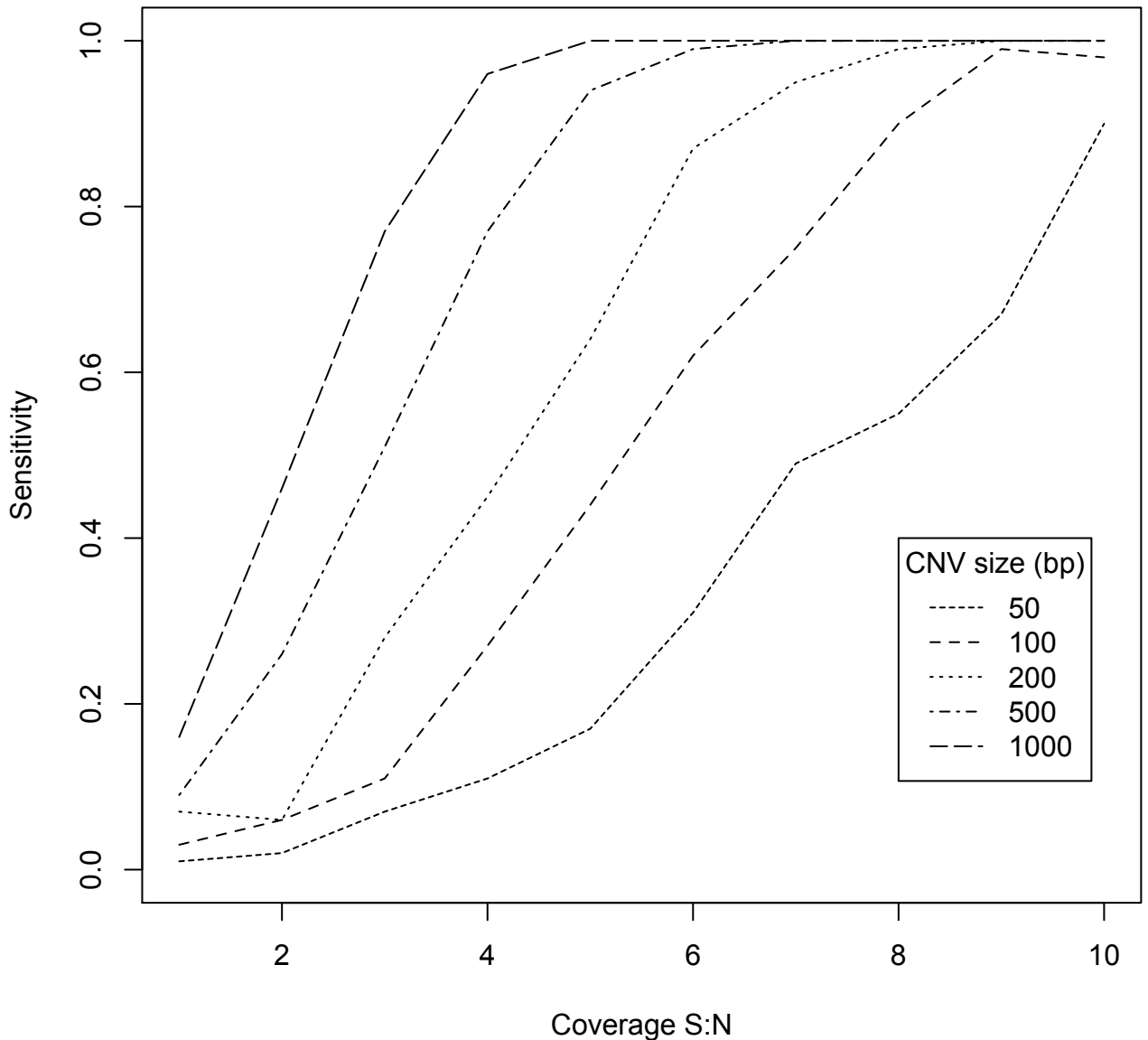

Supplement: Additional file 2 — Figure S2. Simulated sensitivity estimate based on CNV size and signal-to-noise ratio of data. Data simulated for 1 mb of sequence with one true CNV of length 50 bp, 100 bp, 200 bp, 500 bp, or 100 bp. Random noise introduced in sample coverage data at a level corresponding with given signal-to-noise ratio. 100 replications run at signal-to-noise ratios of one to ten for each CNV size. Sensitivity is the proportion of runs in which the CNV was correctly identified. No false positives were identified when signal-to-noise ratio was greater than two (data not plotted). [file 1471-2164-12-184-S2.PDF]

A

**True CNV  
base S:N**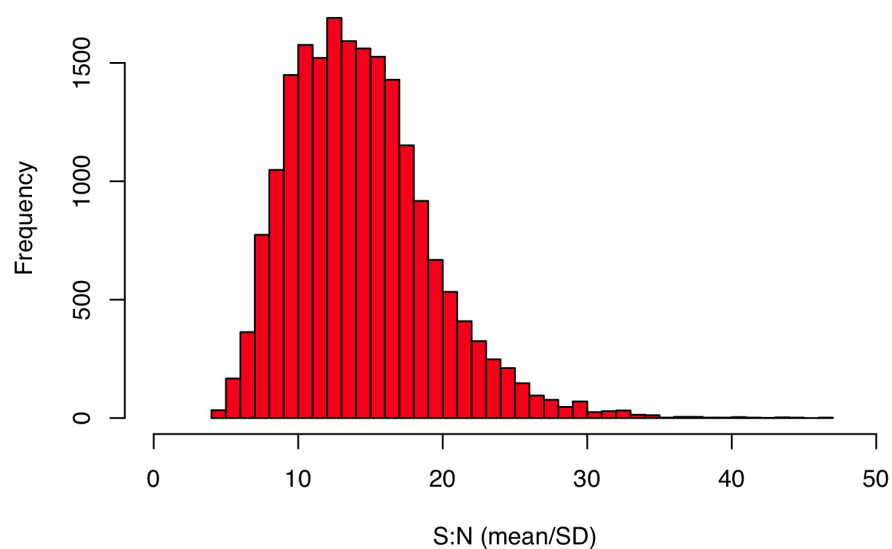**False Positive CNV  
base S:N**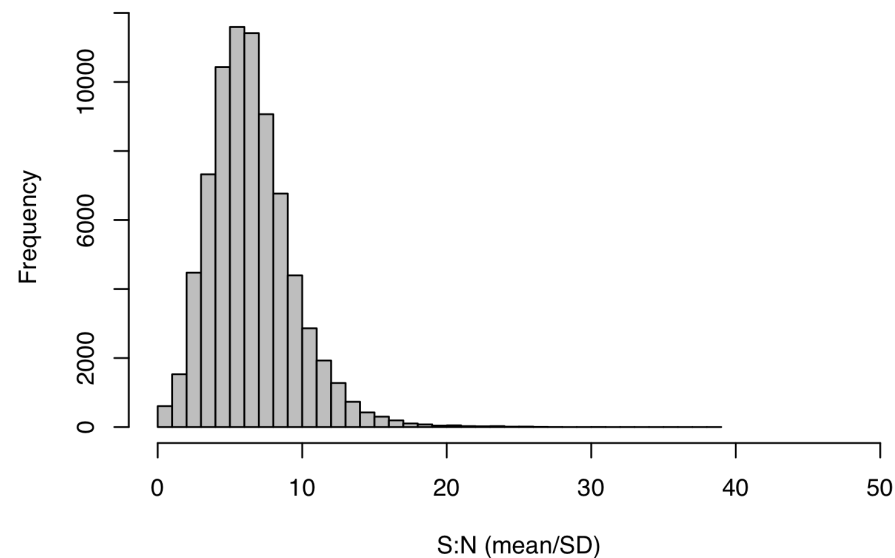

B

**True CNV  
base S:N**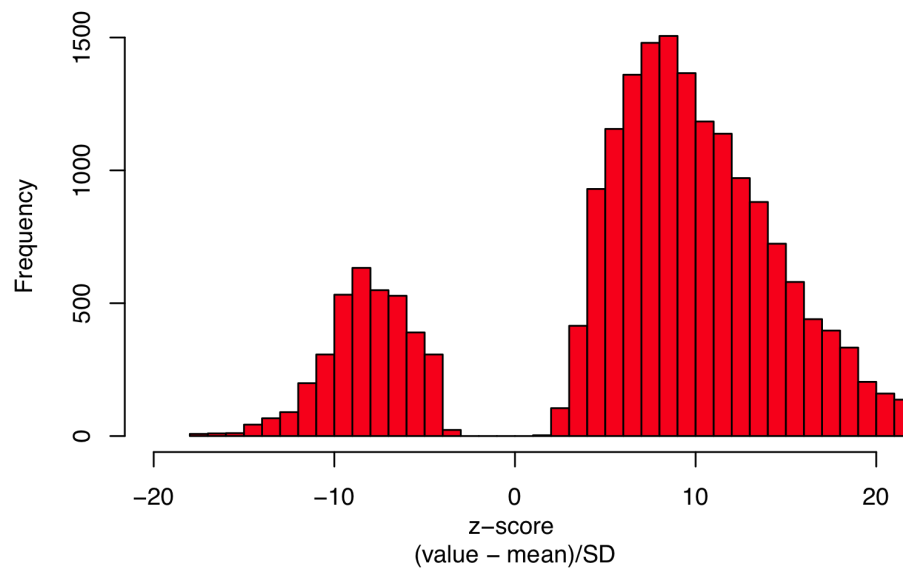**False Positive  
base S:N**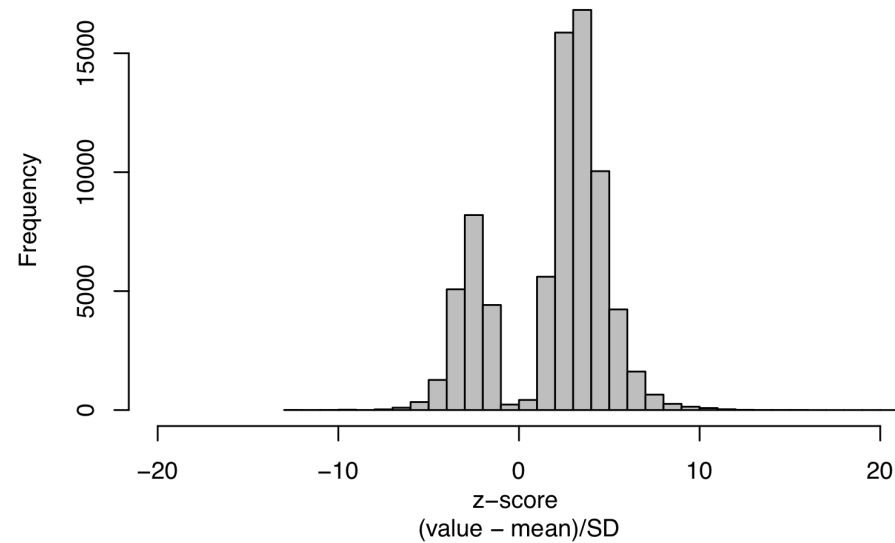

Supplement: Additional file 4 — Figure S3. Comparison of true positive signal and false positive signal. True positive refers to bases within confirmed CNVs, whereas false positive refers to bases with ratio values < 0.6 or >1.4, but where no CNV could be confirmed. Histograms show distribution of: A) S:N (signal to noise: mean/SD), and B) z-score ((value-mean)/SD) for true positive versus false positive bases. [file 1471-2164-12-184-S4.PDF]
